# Global trends in research of pseudomyxoma peritonei: a bibliometric and visualization analysis

**DOI:** 10.3389/fonc.2024.1323796

**Published:** 2024-02-08

**Authors:** Shuo Liu, Xue Liu, Ruiqing Ma, Shuang Yu, Liangyuan Lu, Yanjun Lin, Zhanmin Yang

**Affiliations:** ^1^ Department of Anesthesiology, Aerospace Center Hospital, Beijing, China; ^2^ Department of Endocrinology and Metabolism, Children’s Hospital of Hebei Province, Shijiazhuang, Hebei, China; ^3^ Department of Myxoma, Aerospace Center Hospital, Beijing, China

**Keywords:** pseudomyxoma peritonei, bibliometric analysis, Citespace, VOSviewer, cytoreductive surgery

## Abstract

**Objective:**

Pseudomyxoma peritonei (PMP) was a complex disease that had attracted increasing attention. However, there had been no bibliometric analysis of this disease so far. This study aimed to explore the current situation and frontier trend of PMP through bibliometric and visualization analysis, and to indicate new directions for future research.

**Methods:**

The original research articles and reviews related to the PMP research were downloaded from Web of Science Core Collection on September 11, 2023. CiteSpace (6.2.R4) and VOSviewer(1.6.18) were used to perform bibliometric analysis of the publications, and establish the knowledge map. The data collected was analyzed using the Online Analysis Platform of Bibliometric to evaluate the cooperation of countries in this field.

**Results:**

We identified 1449 original articles and reviews on PMP published between 1998 and 2023. The number of publications on PMP increased continuously. The United States, the United Kingdom and China were the top contributors. The most productive organization was the MedStar Washington Hospital Center. Sugarbaker, Paul H. was the most prolific author and the most cited. Keyword analysis showed that “Pseudomyxoma peritonei”, “cancer”, “cytoreductive surgery”, and “hyperthermic intraperitoneal chemotherapy” were the most common keywords. The earliest and latest used keywords were “mucinous tumors” and “impact”, respectively. “classification”, “cytoreductive surgery”, “appendiceal” were the top 3 strongest citation bursts. The reference “Carr NJ, 2016, *AM J SURG PATHOL*” had the highest co-citations.

**Conclusion:**

This bibliometric analysis showed an increasing trend in literature related to PMP. The research trends and hotspots identified in this study could guide the future research directions in this field, in order to promote the development of PMP.

## Introduction

Pseudomyxoma peritonei (PMP) was a rare and malignant clinical syndrome characterized by widespread spread of copious mucus-containing tumor cells in the abdominal cavity (1, 2). The incidence of PMP was 2-4 per million people and increased with advancing age (2, 3). The incidence of PMP was significantly lower in China and Japan than in the Netherlands, Norway and the United Kingdom (3–6). Women were more likely to develop PMP than men (3). PMP could be secondary to mucinous tumors originating in the abdominal organs, most commonly from the appendix (7, 8). PMP was often named “Jelly-belly” and presented as multifocal mucinous tumors in the abdominal cavity. Due to the accumulation of a large number of mucous tumors, it could lead to increased abdominal circumference, abdominal pain and pressure on internal organs (6). The current standard treatment strategy for PMP was cytoreductive surgery (CRS) combined with hyperthermic intraperitoneal chemotherapy (HIPEC) to further clear microscopic residual tumor (1, 9, 10). This treatment strategy of CRS combined with HIPEC was expected to improve the outcomes of PMP patients, but remained controversial (11–13).

Along with a deeper understanding of PMP, research interest in this subject was growing rapidly. Up to now, researches on PMP had been published in many peer-reviewed journals. But there were still many questions to be resolved. For researchers, quick and accurate knowledge of research hotspots and the latest advances in PMP research would help identify research frontiers and guide clinical practice. However, a quantitative analysis of publications on PMP research was relatively rare.

Bibliometric analysis was a method by quantitative analysis the effects of research outputs (14–17). It could identify potential research collaborations between countries, institutions, or authors in emerging and developing research fields (18, 19). Citation analysis could reflect the influence of a certain author, country or journal in a certain area by analyzing the cited times of articles from a certain author, country or journal by others (20). It was helpful to resolve the current research contradictions, search for new research hotspots, and provide some insights in many areas (21).

This study was expected to develop the knowledge map of PMP from 1998 to 2023 by bibliometric and visualization analysis, which would help researchers and clinicians better grasp the overall trend, comprehend evolution and scientific achievements in PMP field, and provide valuable insights for future research.

## Methods

### Data collection and searching strategies

**Figure 1:**
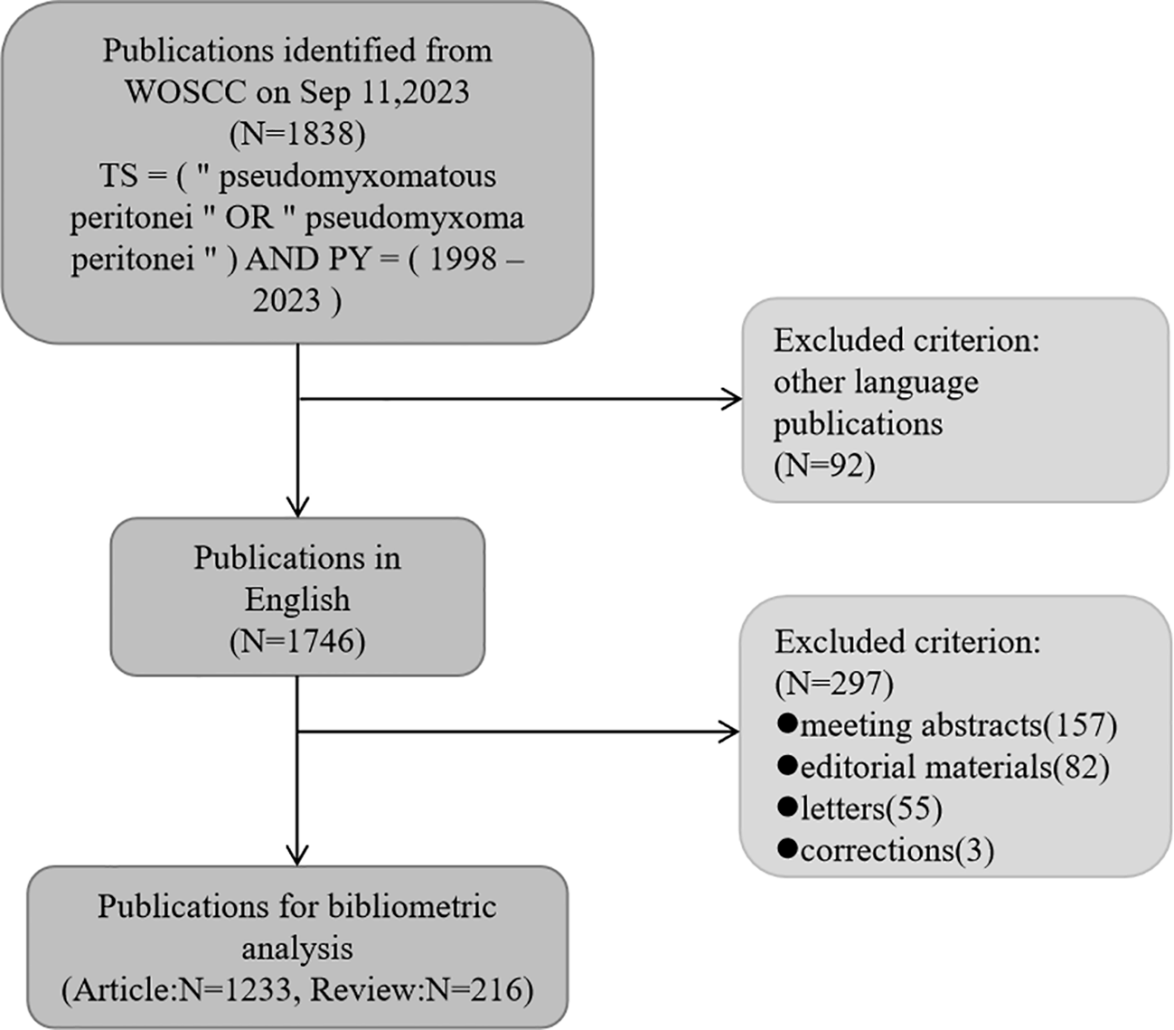
Flow chart of screening publications.

A systematic literature search was performed through the Web of Science Core Collection(WoSCC): TS = (“pseudomyxomatous peritonei” OR “pseudomyxoma peritonei”) AND PY = (1998 – 2023). The document type was limited to original research article and reviews written in English. The literature retrieval and data download were completed on Sep 11, 2023 to avoid bias caused by constant database updates. According to the search strategy, 1449 publications(1233 articles and 216 reviews) were identified for further bibliometric analysis after removing duplicate entries (**Figure 1**). Two researchers conducted the independent literature search. The collaboration between countries was analyzed by the online analysis platform of Bibliometrics (https://bibliometric.com).

### Bibliometric and visualization analysis

CiteSpace (6.2.R4), VOSviewer (1.6.18) and Microsoft Excel 2019 were used to analyze and visualize the literature. A visual network analysis of the collaboration between state institutions and authors was performed by CiteSpace. In the visualization, the node represented an item, such as country, institution, author, keyword, and so on. The co-citation or co-occurrence relationship between nodes was represented by a line, and the thickness of the line was positively correlated with the relationship (22). The concept of intermediate centrality proposed by American sociologist Freeman was adopted to determine the pivot point (23). The betweenness centrality could be used to evaluate the importance of each publication at the level of the co-citation network (24). VOSviewer could display scientific knowledge maps, density color visualization, project cluster analysis and many other bibliometric graphs (25). Microsoft Excel 2019 was used to analyze the polynomial function of the prediction model, and the time trends of publication and citation.

## Results

### Growth trend of publications

**Figure 2:**
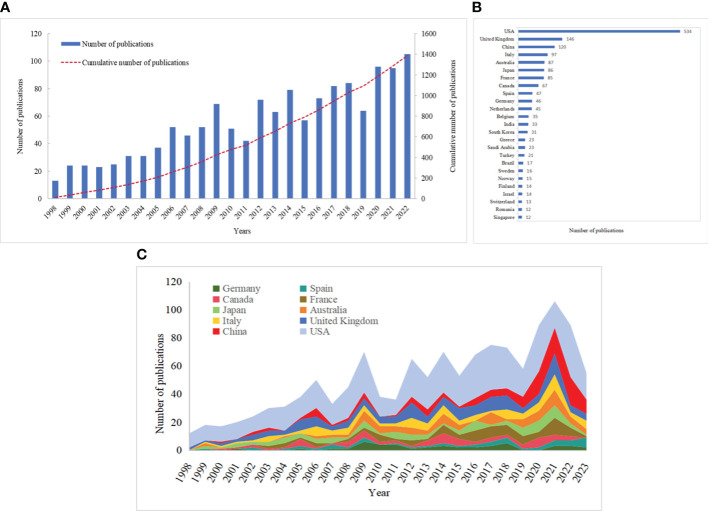
Annual publications; numbers of publications by countries/regions. **(A)** Annual number and cumulative number of publications worldwide. **(B)** Top 25 countries/regions with more than 10 of PMP publications. **(C)** The number of annual publications of the top 10 countries/regions for PMP publications.

By our search strategy, we collected 1449 publications between 1998 and 2023, including 1233 research articles and 216 reviews. These papers regarding PMP had been cited a total of 40041 times, with an average of 27.63 citations and an H-index of 94 (September 11, 2023). To investigate developments about PMP, we plotted the number of publications each year (**Figure 2A**). It showed that the number of publications increased steadily per year, reflecting the growing interest of researchers in PMP. Based on the highest R^2^, the polynomial function predicted that 119 articles on PMP would be published in 2025(y = 0.006x^3^ - 0.2262x^2^ + 5.5989x + 7.3957, R² = 0.8915).

The included publications were divided into five sub-categories by Web of Science Categories: surgery, oncology, pathology, biology and others. We plotted the number of publications by categories (**Supplementary Figure 1**). It showed that the number of publications in PMP was increasing year by year in all sub-categories. The number of papers in the surgery and oncology increased significantly. It was worth noting that not until about 2014, the biological papers on PMP were published steadily every year. This showed that researchers were gradually paying attention to the biological study of PMP in recent 10 years.

### Countries/regions and organizations analysis

These publications about PMP came from 1485 organizations in 64 countries/regions. Countries/regions with more than 10 publications were shown in **Figure 2B**. The United States was the most published country, with 534 publications, followed by the United Kingdom(144), China(120), Italy(97), Australia(87). **Table 1** and **Figure 2C** showed the top 10 countries/regions and their numbers of publications each year, respectively. The publications about PMP in these countries were steadily increasing. However, citation analysis showed that France had the highest average article citation(46.71), in the top 10 productive countries (**Table 1**). Although China contributed a lot in the number of publications, it still needed to improve in the average article citation.

**Table 1:** The top 10 countries/regions of publications on PMP.

Rank	Country/region	Publications	Citations	Average article citations
1	USA	534	21387	40.0505618
2	United Kingdom	146	5318	36.42465753
3	China	120	882	7.35
4	Italy	97	3507	36.15463918
5	Australia	87	3040	34.94252874
6	Japan	86	1178	13.69767442
7	France	85	3970	46.70588235
8	Canada	67	2527	37.71641791
9	Spain	47	1911	40.65957447
10	Germany	46	1802	39.17391304

**Figure 3:**
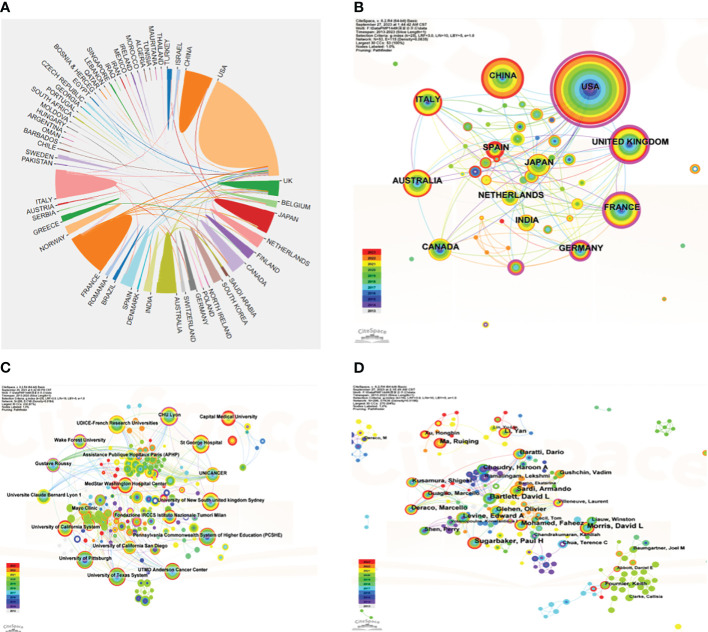
Collaboration relationship among countries/regions, institutions, and authors involved in PMP research. **(A)** Collaborations between countries/regions on PMP. The size of the plates indicates the number of publications in corresponding country/region, and the lines between the plates indicate the intensity of collaboration between them. **(B)** Visualization of country/regional cooperation networks. N = 53, E = 115. **(C)** Visualization of organizational cooperation networks. N = 286, E = 748. **(D)** Visualization of author cooperation networks. N = 286, E = 636. The size of the circle reflects the frequency of co-occurrence, and the thickness of the line indicates the co-occurrence relationship. (N: Number of nodes. E: Number of links.).

Cooperation between countries/regions in PMP research was shown in **Figure 3A**. The United States had the most cooperation with other countries. In the VOSviewer citation network map, the purple circle represented the betweenness centrality of this node was greater than 0.1. As shown in the **Figure 3B**, the United States, France and the United Kingdom had high betweenness centralities. It meant that they were very important in this field of PMP and had much more influence.

The top 10 organizations by publications on PMP research were listed in **Table 2**. Most of them were from the United States, with the rest from Australia, France and Italy. The most productive organizations were the Medstar Washington Hospital Center(93), followed by the University of New South Wales(68), the University of Texas System(46). Considering the name change and reorganization of institutions, we analyzed the different names of the same organizations included in this study and listed the different names of the top 10 productive organizations in **Supplementary Table 1**. Visualization of organizational collaboration networks was mapped by CiteSpace with 286 organizations and 748 collaborations (**Figure 3C**). It showed that CHU Lyon, Gustave Roussy, and Wake Forest University were very active in collaboration with other institutions. The Gustave Roussy showed the highest centrality(0.16).

**Table 2:** The top 10 organizations, authors of publications on PMP.

Rank	Organization	Publications	Citations	Author	Publications	Citations
1	Medstar Washington Hosp Ctr	93	6686	Sugarbaker, Paul H.	92	5860
2	Univ New South Wales	68	2833	Morris, David L.	50	2109
3	University of Texas System	46	1431	Levine, Edward A.	35	1918
4	Univ Pittsburgh	44	1288	Baratti, Dario	33	2055
5	FDN IRCCS Ist Nazl Tumori	43	2778	Deraco, Marcello	33	2037
6	Wake Forest Univ	43	2547	Bartlett, David L.	32	867
7	CHU Lyon	39	2090	Kusamura, Shigeki	31	1328
8	University of California System	36	963	Chua, Terence C.	30	1852
9	University Claude Bernard Lyon 1	33	1177	Mohamed, Faheez	30	1225
10	UNICANCER	33	2347	Sardi, Armando	29	1223

A total of 5988 authors had made significant contributions to PMP research. Sugarbaker, Paul H. was the most productive author with 92 publications, followed by Morris, David L.(50), Levine, Edward A. (35). Sugarbaker, Paul H. was also the most cited author(5860) (**Table 2**). However, as shown in **Figure 3D**, Sugarbaker Paul H cooperated closely with Levine Edward A, but not with Morris David L. Villeneuve Laurent had the highest centrality(0.13), and he also collaborated closely with Sugarbaker Paul H.

### Analysis of journals and co-cited journals

A total of 356 journals contributed to PMP publications, with 31 journals publishing at least 10 papers. The top 10 journals and co-citations were showed in **Table 3**. Six of the top 10 productive journals were classified as Q1 JCR division, according 2023 edition of the Journal Citation Reports (JCR), indicating that these journals had high credibility and quality. The top 3 productive journals were Annals of Surgical Oncology(Publications=164, IF=3.7), EJSO(Publications=79, IF=3.8), Journal of Surgical Oncology(Publications=54, IF=2.5). However, the journal with the highest IF was British Journal of Surgery(IF=9.6, Q1).

**Table 3:** The top 10 productive and co-cited journals.

Rank	Journals	Publications	Citations	IF (2023)	JCR	Co-cited journal	Citations	IF (2023)	JCR
1	Ann Surg Oncol	164	5741	3.7	Q1	ANN SURG Oncol	4493	3.7	Q1
2	EJSO	79	1965	3.8	Q1	Am J Surg Pathol	2750	5.6	Q1
3	J Surg oncol	54	1385	2.5	Q2	Ann Surg	2145	9.0	Q1
4	World J Surg Oncol	29	460	3.2	Q2	J Clin Oncol	1697	45.3	Q1
5	Am J Surg Pathol	26	3347	5.6	Q1	J Surg oncol	1521	2.5	Q2
6	Int J Hyperther	23	407	3.1	Q2	EJSO	1494	3.8	Q1
7	Dis Colon Rectum	21	786	3.9	Q1	CANCER	1285	5.2	Q1
8	Human Pathology	20	568	3.3	Q2	Brit J Surg	1170	9.6	Q1
9	Brit J Surg	17	1529	9.6	Q1	Cancer-Am Cancer Soc	922	6.2	Q1
10	CANCER	16	35	5.2	Q1	Dis Colon Rectum	906	3.9	Q1

**Figure 4:**
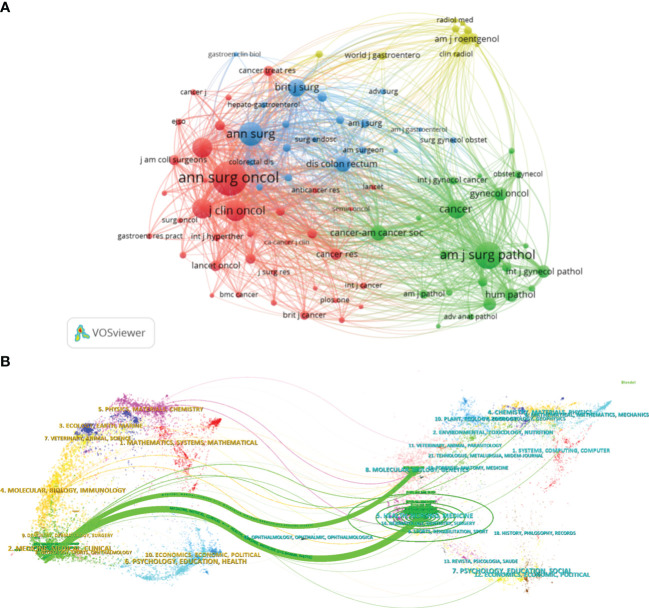
Visualization of the journal co-occurrence network and the dual map overlay of journals on PMP. **(A)** The different colors indicated various clusters. The strongest co-citation links between journals are represented by lines. **(B)** Dual map overlay of journals. Citing journals are presented on the left, cited journals are on the right, and the colored paths represent the citation relationships.

As shown in **Table 3**, the top 3 journals with the highest citations were Annals of Surgical Oncology(5741 citations), American Journal of Surgical Pathology(3347 citations), EJSO(1965 citations). Meanwhile, Annals of Surgical Oncology was also the journal of the highest co-citation(4493 co-citations) (**Figure 4A**). CiteSpace was used to visualize the dual map overlay of journals to illustrate the distribution of academic journals (26). As shown in **Figure 4B**, the left half of the graft represented the citing journals, and the other half symbolized cited journals. The colored line represented the primary citation pathway, indicating that PMP research in health, nursing, medicine journals(z=5.64) and molecular, biology, genetics journal(z=1.96) were primarily cited by publications in medicine, medical, and clinical journals, respectively.

### Keyword co-occurrence analysis of PMP research hotspots

**Figure 5:**
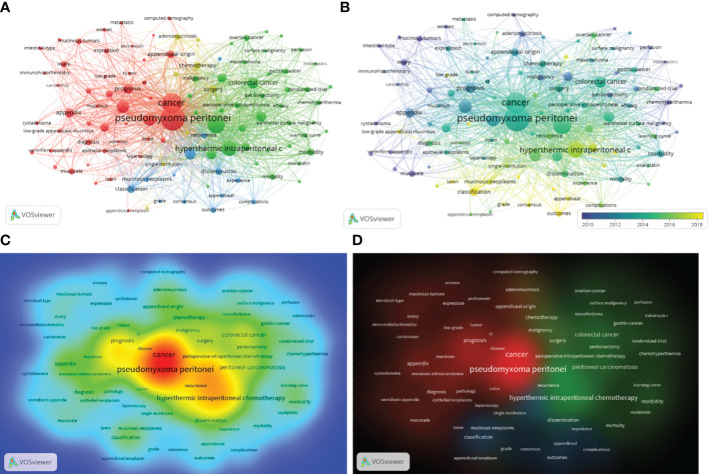
The network visualization, overlay visualization, and density visualization of keywords for PMP. **(A)** The visualization of the co-occurrence network of keywords for PMP publications. The 110 keywords were divided into 4 clusters in red, green, yellow, blue. Each cluster represents a research direction; the size of the circle inside the cluster represents the number of occurrences; and the thickness of the lines indicates the the strength of the connection. **(B)** Visualization of overlay analysis of keyword co-occurrence network. The overlay visualization of keyword co-occurrence colors the different clusters according to the timeline. The purple nodes represents that the keywords have been used in early publications. As time passes, the yellow nodes indicate that the keywords have been commonly used in recent publications. **(C)** The item density visualization map. The red nodes represent commonly used keywords. **(D)** The cluster density visualization map. The depth of the color node represents commonly used keywords.

In order to observe the hot spots and emerging trends in PMP research, we analyzed the occurrence frequency and co-occurrence of keywords (27, 28). In total, 2964 keywords were extracted from 1449 publications. **Table 4** demonstrated the top 20 frequent keywords. “Pseudomyxoma peritonei”, “cancer”, “cytoreductive surgery”, and “hyperthermic intraperitoneal chemotherapy” appeared in more than 400 papers, indicating that surgery and hyperthermic intraperitoneal chemotherapy were the primary treatment methods and research trends of PMP, at present. VOSviewer showed the network visualization built with 110 keywords with more than 20 occurrences (**Figure 5A**). There were 4 major clusters identified by different colors. They reflected the main topics of PMP, including “pseudomyxoma peritonei”, “treatment”, “survival”, and “adenomucinosos”.

**Table 4:** Top 20 keywords in PMP publications.

Rank	Keyword	Occurrences	Total link strength
1	pseudomyxoma peritonei	1130	8460
2	cancer	761	5941
3	cytoreductive surgery	584	5050
4	hyperthermic intraperitoneal chemotherapy	424	3692
5	origin	305	2553
6	clinicopathological analysis	295	2634
7	survival	229	1964
8	colorectal cancer	224	2055
9	peritoneal carcinomatosis	199	1769
10	prognosis	181	1627
11	management	176	1536
12	appendix	165	1252
13	intraperitoneal chemotherapy	150	1272
14	systemic chemotherapy	134	1280
15	adenocarcinoma	132	1140
16	classification	126	1001
17	appendiceal origin	111	929
18	morbidity	99	895
19	surgery	99	824
20	features	97	833

The overlay visualization of 110 keywords showed the research trends of PMP during different periods (**Figure 5B**). The keyword of the earliest research topic were “mucinous tumors”(67publications, average publication year: 2006.72), but recently the keywords of the most common research topic was “impact”(24publications, average publication year: 2020.50). The frequency of keywords occurrence was shown by the density visualization (**Figures 5C, D**), which indicated that “pseudomyxoma peritonei”, “cancer”, “recurrence”, “hyperthermic intraperitoneal chemotherapy” were most commonly used in PMP publications.

### Time evolution analysis and citation burstiness analysis of keywords in PMP publications

**Figure 6:**
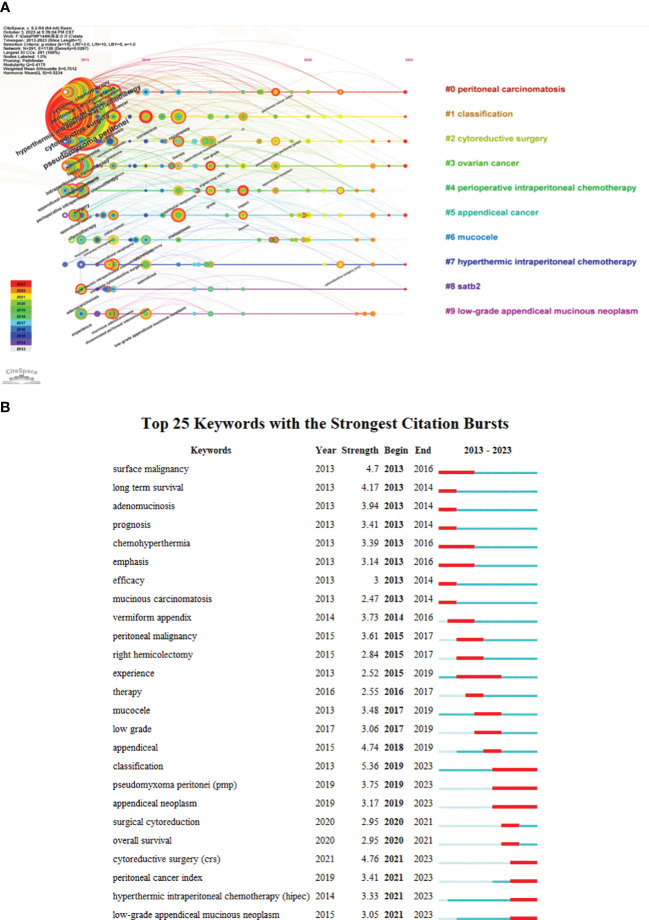
Timeline view of keyword co-occurrence and keywords with the strongest citation bursts in PMP publications. **(A)** Visualization map of timeline viewer related to PMP research. N = 291, E = 1126. **(B)** The top 25 keywords with the strongest citation bursts in PMP publications. The length of the red line indicates the duration of the burst. The burst intensity represents that the keyword is the main hotspot in the research of PMP.

CiteSpace was used to analyze keywords co-occurrence in PMP research. The timeline view was mapped in **Figure 6A**, which had 291 keywords and 1126 co-occurrence relationships. As can be seen from the time line view, “peritoneal carcinomatosis” was the largest cluster, followed by “classification”, and “cytoreductive surgery”. Seven of them were still in progress, except for “perioperative intraperitoneal chemotherapy”, “mucocele”, and “low-grade appendiceal mucinous neoplasm”.

Keyword burst was a bibliometric algorithm, indicating possible hot research topics of a specific research field (29). From 1998 to 2023, “classification”, “cytoreductive surgery”, “appendiceal” were the top 3 strongest citation bursts, with the strength values of 5.36, 4.76, 4.74, respectively (**Figure 6B**). “Classification” was also the strongest citation bursts since 2019. Among the top 25 keywords with the strongest citation bursts, 7 were still the research hot spots, indicating that the current research on PMP involved many perspectives.

### Co-citation and clustered visualization network of co-cited reference

**Figure 7:**
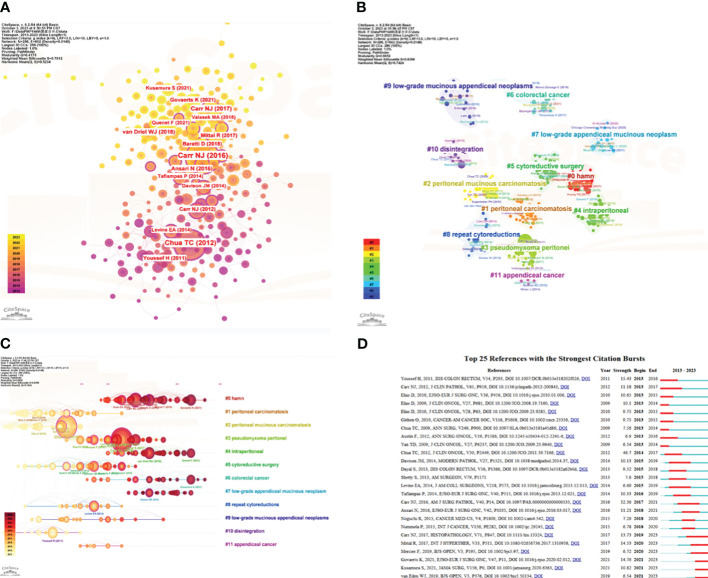
The co-cited reference clustering and strongest bursts of co-cited reference. **(A)** Visualization map of co-citation of references on PMP. Nodes marked with purple circles (centrality >0.1) are significant in this co-citation visualization network map. **(B)** Clustering network visualization of co-cited references. **(C)** Timeline view of co-citation clusters. **(D)** The top 25 publications with the strongest citation bursts of co-cited publications on PMP research. The length of the red line indicates the duration of the burst. The burst strength represents that the reference is of great significance to the research of PMP.

The co-citation and clustering network analysis of 17389 citations were carried out by CiteSpace. Clustering was efficient and compelling (S score=0.701, Q score = 0.418). As shown in **Figure 7A**, the visual network map of co-cited publications had 286 nodes and 602 links. Among the co-citations PMP publications, the top 3 with the highest centrality were “Chua TC, 2012” (Centrality=0.21) (30), “Levine EA, 2014” (centrality=0.2) (31) and “Carr NJ, 2012” (centrality=0.17) (32). **Table 5** listed the top 10 highly co-cited publications. Carr NJ in American Journal of Surgical Pathology(167) (33) got the most co-cited publications, followed by Chua TC in Journal of Clinical Oncology(146) (30). **Figure 7B** showed the 12 main clusters of the co-cited PMP publications, marked with different colors. Similarly, in **Figure 7C**, the node site on the time axis represented the time of reference, and the node size denoted the total citations of reference. “HAMN”, and “colorectal cancer” were recent clusters, representing the current research hotspots of PMP.

**Table 5:** The top 10 co-cited publications in PMP from 1998 to 2023.

Rank	Title	First author	Year	Journal	Cited frequency	DOI
1	A Consensus for Classification and Pathologic Reporting of Pseudomyxoma Peritonei and Associated Appendiceal Neoplasia: The Results of the Peritoneal Surface Oncology Group International (PSOGI) Modified Delphi Process	Carr NJ	2016	AM J SURG PATHOL	167	10.1097/PAS.0000000000000535
2	Early- and long-term outcome data of patients with pseudomyxoma peritonei from appendiceal origin treated by a strategy of cytoreductive surgery and hyperthermic intraperitoneal chemotherapy	Chua TC	2012	J CLIN ONCOL	146	10.1200/JCO.2011.39.7166
3	The histopathological classification, diagnosis and differential diagnosis of mucinous appendiceal neoplasms, appendiceal adenocarcinomas and pseudomyxoma peritonei	Carr NJ	2017	HISTOPATHOLOGY	59	10.1111/his.13324
4	Cytoreductive surgery and hyperthermic intraperitoneal chemotherapy in 1000 patients with perforated appendiceal epithelial tumours	Ansari N	2016	EJSO-EUR J SURG ONC	45	10.1016/j.ejso.2016.03.017
5	Hyperthermic Intraperitoneal Chemotherapy in Ovarian Cancer	van Driel WJ	2018	NEW ENGL J MED	44	10.1056/NEJMoa1708618
6	Operative findings, early complications, and long-term survival in 456 patients with pseudomyxoma peritonei syndrome of appendiceal origin	Youssef H	2011	DIS COLON RECTUM	42	10.1007/DCR.0b013e318202f026
7	Pseudomyxoma peritonei: natural history and treatment	Mittal R	2017	INT J HYPERTHER	41	10.1080/02656736.2017.1310938
8	Pathology and prognosis in pseudomyxoma peritonei: a review of 274 cases	Carr NJ	2012	J CLIN PATHOL	40	10.1136/jclinpath-2012-200843
9	Validation of the Recent PSOGI Pathological Classification of Pseudomyxoma Peritonei in a Single-Center Series of 265 Patients Treated by Cytoreductive Surgery and Hyperthermic Intraperitoneal Chemotherapy	Baratti D	2018	ANN SURG ONCOL	40	10.1245/s10434-017-6252-1
10	Appendiceal tumours and pseudomyxoma peritonei: Literature review with PSOGI/EURACAN clinical practice guidelines for diagnosis and treatment	Govaerts K	2021	EJSO-EUR J SURG ONC	38	10.1016/j.ejso.2020.02.012

Professor Chen Chaomei suggested that references with strong value in the strength column tend to be the milestone in scientific cartographic research (34). As can be seen from the top 25 references with strongest citation bursts between 2013 to 2023, it was an important milestone in the development of PMP research (**Figure 7D**). The strongest burst reference(strength=46.7) was titled “Early- and long-term outcome data of patients with pseudomyxoma peritonei from appendiceal origin treated by a strategy of cytoreductive surgery and hyperthermic intraperitoneal chemotherapy” (30), published in Journal of Clinical Oncology by Chua TC et al. in 2012, with citation bursts from 2014 to 2017. There were 5 references-maintained citation peaks until 2023, which referred to the classification and outcomes of PMP, indicating that these were still hot research spots for current.

## Discussion

Bibliometric analysis could help researchers to get the research hotspot and predict the future development trend in a certain field (35). However, there were few bibliometric analysis articles on the PMP. In this study, we conducted a comprehensive bibliometric analysis of studies on PMP by the VOSviewer and CiteSpace software. We performed the burst hotspot analysis, cluster analysis, and keyword analysis to identify the current research emphases and development trend of PMP. Based on the data published in the WoSCC database between January 1, 1998 and September 11, 2023, we identified 1449 original articles and reviews. The number of publications about PMP had grown every year during the last 25years. And this suggested that PMP will continue to receive the attention of researchers in the foreseeable future.

The analysis of the number of publications by country showed significant differences between countries, with only the United States, the United Kingdom, and China publishing more than 100 articles. That may be related to the significant financial support of the three countries in this medical field. It was worth noting that the number of publications in China had increased significantly in recent years, which indicated that more and more Chinese researchers were interested in this field. In the national cooperation networks, the United States had the most extensive cooperation network. It illustrated that the United States occupied an important position in PMP research. We should make more cooperation with them in PMP field. However, for China, there was less international cooperation, indicating that strengthening international cooperation contributed to the PMP research of China.

Research on the number of institutional publications showed that most of organizations contributed to PMP were from the United States. However, the analysis of co-cited authors showed that Norman J Carr, the most cited author(167citations), was from Peritoneal Malignancy Institute, Basingstoke, the United Kingdom. The second most co-cited author was Terence C. Chua from University of New South Wales, St George Hospital, Sydney, Australia. This indicated that they had relatively authoritative academic status in this field.

The publication and co-citation analysis of journal could provide suggestions for researchers to select appropriate journal for submission. Their research results could be successfully accepted and contribute to the research progress in this field. These papers were mainly published in Q1 journals. And the co-cited journals were also mainly located in Q1 region. The journal with the highest citation and co-citation was Annals of Surgical Oncology(IF=3.7, Q1), which was also the journal with the highest number of publications. This indicated that it had high authority and credibility in the field of PMP. The match rate between the top 10 most cited and most prolific journals was 70%, suggesting that the quantity and quality of PMP research was not yet balanced. It was necessary to reinforce international cooperation among researchers to improve the quality of research.

Among the top 10 co-cited references, the most cited one was a consensus about classification and pathologic reporting of the PMP (33). PMP was a peritoneal malignancy characterized by mucous ascites and peritoneal implants that usually originate in the appendix (36). The clinical manifestations and pathological characteristics of PMP were varied and unpredictable (1). So, the pathologic classification criteria and diagnostic terms of PMP were confusing and had been the research focus for a long time. There were many classification systems, such as Ronnett three-layer classification (37), Bradley two-layer classification (38), and WHO two-layer classification (39). As for the classification of PMP, it related to the diagnosis and prognosis of PMP. It was not until 2016 that a consensus was reached on the pathology classification and diagnostic terminology of PMP (33). In this consensus, PMP was divided into four categories: low grade, high grade, and high grade with signet ring cells, and acellular mucin classified separately (33). And it was confirmed that patients with acellular mucin had a good prognosis, while patients with high grade signet ring cells had a very poor prognosis (40). This classification was still recommended in the 2021 guidelines for diagnosis and treatment of PMP (36).

The second most cited was a multi-institutional registry study on the outcome of PMP undergoing CRS combined with HIPEC, including early and long-term outcomes (30). It showed that the incidence of postoperative complications and mortality in PMP patients after CRS combined with HIPEC were acceptable. 63% of patients survived for more than 10 years. It also suggested that debulking surgery and chemotherapy treatments before definitive CRS may not contribute to surgical success and outcomes (30). And the optimal cytoreductive surgery could improve the outcomes of PMP patients (30). At present, although consensus had been reached in terms of pathological classification, terminology, CRS technical details, there was still controversy about HIPEC protocol (1).

The keywords of a scientific article could reflect the overall topic of the study. So the analysis of the frequency and co-occurrence of keywords contributed to identify the important topic and trends in a particular field of study (28). **Table 4** and **Figure 5** showed the frequently-used keywords in PMP research, including pseudomyxoma peritonei, cancer, cytoreductive surgery, hyperthermic intraperitoneal chemotherapy, origin, clinicopathological analysis, survival, et al. With the in-depth study of PMP, new topics emerge endlessly, which reflected the future research trend. PMP was a rare clinically malignant syndrome with an incidence of 2-4 per million (1). Before 2010, the keywords mainly represent as mucinous tumors, ovary and women. Previous studies of PMP focused on clinical features. After a long time of clinical practice to further understand the pathogenesis of PMP, the emerging keywords such as “low-grade appendiceal mucinous neoplasm”, “CRS”, and “HIPEC” possessed the strongest citation bursts. It showed that researchers played more attention to the classification and treatment on PMP. The same conclusion is also observed in cluster analysis of PMP.

CRS combined with HIPEC was the recommended treatment for PMP (1). It was first reported in 1980 (41). Standardized CRS, which referred to the complete removal of all visible malignancies, was the basis for long term survival (42). Because of the collaboration between research institutions, surgical procedures and technical details had been improved and standardized (1). In contrast, there were some controversies need to be resolved in HIPEC. Most of the commonly used HIPEC regimens were based on oxaliplatin and mitomycin C (1). However, the use of these two drugs was controversial because high doses could cause serious complications (12, 31). In 2018, a multicenter randomized controlled trial comparing mitomycin to oxaliplatin HIPEC for appendiceal cancer showed that both of them were associated with mild blood toxicity, but mitomycin had slightly higher hepatotoxicity than oxaliplatin (43).

In this study, we analyzed the publications on PMP through bibliometric analysis and found that most of them mainly focused on clinical studies, such as classifications, treatments, outcomes, and immune markers(like SATB-2) (44, 45). From the above analysis, it could be seen that there were few basic researches on PMP. We should actively search for the pathogenesis and metastatic mechanism of PMP, to fundamentally inhibit and alleviate PMP. In addition, it was important to actively search for the methods and potential indicators to diagnose PMP earlier, in order to select the most optimal treatment strategy for patient. Currently, there were no effective molecular or pathway-targeted interventions to treat PMP. In the future, more research funding was needed for the basic research.

## Limitations

Although bibliometrics analysis was more advantageous in analyzing research hotspots and trends in a certain field, which was more objective and quantitative. However, this study still had limitations. Only the publications reported in English language were included. Some non-English language literature on PMP was ignored. But the numbers of them were relatively small. In addition, due to the limitations of CiteSpace software, the publications only from WoSCC were analyzed. Other databases such as Embase, Scopus and PubMed were not included in this study. It was noteworthy that the WoSCC was the most commonly used scientometrics database.

## Conclusion

This study provided some insights on PMP for researchers and clinicians through bibliometrics and visualization analysis. It summarized the research trends of PMP in recent 25 years. The numbers of publications were also increasing year by year in PMP field. This research could help researchers locate important collaborators and literature, provide recommendations for publishing journals, and discover research hotspots. The analysis reviewed significant contributions to the PMP and encouraged more research in the scientific community.

## Data availability statement

The original contributions presented in the study are included in the article/**Supplementary Material**. Further inquiries can be directed to the corresponding author.

## Author contributions

SL: Writing – original draft. XL: Software, Writing – review & editing. RM: Writing – review & editing. SY: Writing – review & editing. LL: Writing – review & editing. YL: Writing – review & editing. ZY: Writing – review & editing.
