# Supplementary figures and images for: Global trends in research of pseudomyxoma peritonei: a bibliometric and visualization analysis
Source: Front Oncol. 2024 Feb 8;14:1323796. doi: 10.3389/fonc.2024.1323796 (PMC10883648; doi:10.3389/fonc.2024.1323796)

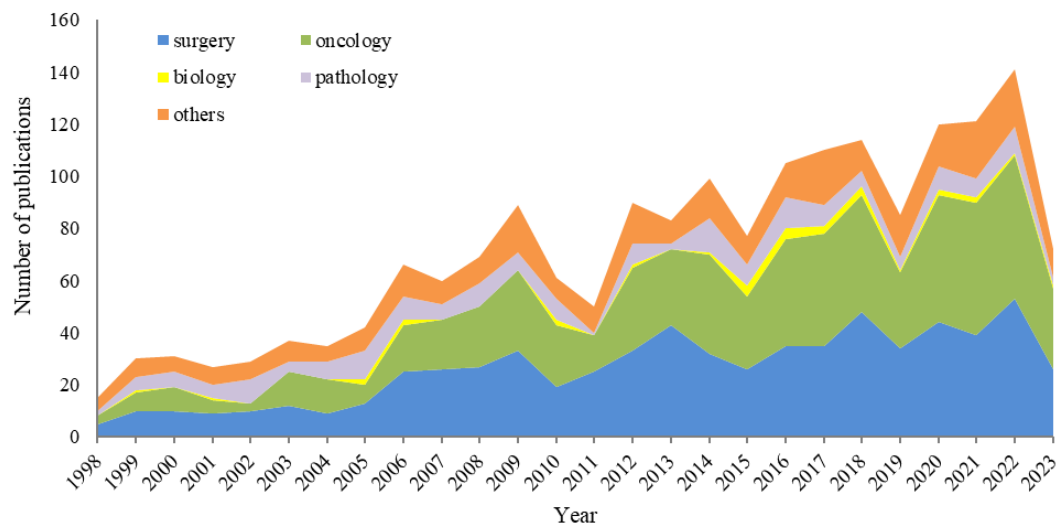

Supplementary fig. 1. Number of publications by categories

Supplement: Supplementary file 1 [file Image_1.pdf]
