# Supplementary material for: Global trends in research of pseudomyxoma peritonei: a bibliometric and visualization analysis
Source: Front Oncol. 2024 Feb 8;14:1323796. doi: 10.3389/fonc.2024.1323796 (PMC10883648; doi:10.3389/fonc.2024.1323796)
Supplement: Supplementary file 2 [file Table_1.docx]

Supplementary table 1: The top 10 organizations of publications on PMP

| Rank | Organization | Publications^+^ | Citations^++^ | The different names of the same organization* | Publications | Citations |
| --- | --- | --- | --- | --- | --- | --- |
| 1 | MedStar Washington Hospital Center | 93 | 6686 | Washington Cancer Institute | 52 | 3307 |
|  |  |  |  | Washington Hospital Center | 47 | 3496 |
|  |  |  |  | MedStar Washington Hospital Center | 23 | 289 |
| 2 | University of New South Wales Sydney | 68 | 2833 | University of New South Wales | 57 | 2638 |
|  |  |  |  | St. George Hospital | 37 | 583 |
| 3 | University of Texas System | 46 | 1431 | University of Texas MD Anderson Cancer Center | 32 | 979 |
|  |  |  |  | University of Texas | 10 | 399 |
|  |  |  |  | University of Texas Houston | 3 | 74 |
|  |  |  |  | University of Texas Southwestern Medical Center Dallas | 2 | 39 |
| 4 | University of Pittsburgh | 44 | 1288 | University of Pittsburgh | 44 | 1288 |
| 5 | Fondazione IRCCS Istituto Nazionale Tumori Milan | 43 | 2778 | National Cancer Institute | 23 | 2278 |
|  |  |  |  | Fondazione IRCCS Istituto Nazionale dei Tumori | 20 | 500 |
| 6 | Wake Forest University | 43 | 2547 | Wake Forest University | 27 | 2199 |
|  |  |  |  | Wake Forest Baptist Health | 11 | 234 |
|  |  |  |  | Wake Forest School of Medicine | 8 | 164 |
| 7 | CHU Lyon | 39 | 2090 | Hospices Civils de Lyon | 25 | 1285 |
|  |  |  |  | Centre Hospitalier Lyon Sud | 21 | 667 |
|  |  |  |  | CHU Lyon Sud | 4 | 459 |
| 8 | University of California system | 36 | 963 | University of California San Diego | 26 | 508 |
|  |  |  |  | University of California San Francisco | 6 | 292 |
|  |  |  |  | University of California Irvine | 2 | 0 |
|  |  |  |  | University of California Los Angeles | 2 | 163 |
| 9 | University Claude Bernard Lyon 1 | 33 | 1177 | University Claude Bernard Lyon 1 | 33 | 1177 |
| 10 | UNICANCER | 33 | 2347 | Institute Gustave Roussy | 31 | 2339 |
|  |  |  |  | Centre Val d'Aurelle | 4 | 704 |
|  |  |  |  | Centre Léon Bérard | 2 | 18 |
|  |  |  |  | Institute Curie | 2 | 15 |

^+^, ^++^: They were the results of de-duplicating and counting publications after consolidating different names of the same organization.

*: The organization published more than 1 paper under this name.
